# A robust 6-mRNA signature for prognosis prediction of pancreatic ductal adenocarcinoma

**DOI:** 10.7150/ijbs.32899

**Published:** 2019-08-22

**Authors:** Chenhao Zhou, Yue Zhao, Yirui Yin, Zhiqiu Hu, Manar Atyah, Wanyong Chen, Zhefeng Meng, Huarong Mao, Qiang Zhou, Weiguo Tang, Pengcheng Wang, Zhanming Li, Jialei Weng, Christiane Bruns, Marie Popp, Felix Popp, Qiongzhu Dong, Ning Ren

**Affiliations:** 1 Department of Liver Surgery, Liver Cancer Institute, Zhongshan Hospital, Fudan University and Key Laboratory of Carcinogenesis and Cancer Invasion, Ministry of Education, Shanghai, China;; 2 Department of General, Visceral and Cancer Surgery, University Hospital of Cologne, Cologne, Germany;; 3 Department of Surgery, Otto-von-Guericke University, Magdeburg, Germany;; 4 Institute of Fudan Minhang Academic Health System, Minhang Hospital, Fudan University, Shanghai, China;; 5 Institutes of Biomedical Sciences, Fudan University, Shanghai, China.

**Keywords:** Pancreatic ductal adenocarcinoma, molecular signature, survival

## Abstract

Pancreatic ductal adenocarcinoma (PDAC) is one of the most fatal malignancies worldwide. PDAC prognostic and diagnostic biomarkers are still being explored. The aim of this study is to establish a robust molecular signature that can improve the ability to predict PDAC prognosis. 155 overlapping differentially expressed genes between tumor and non-tumor tissues from three Gene Expression Omnibus (GEO) datasets were explored. A least absolute shrinkage and selection operator method (LASSO) Cox regression model was employed for selecting prognostic genes. We developed a 6-mRNA signature that can distinguish high PDAC risk patients from low risk patients with significant differences in overall survival (OS). We further validated this signature prognostic value in three independent cohorts (GEO batch, *P* < 0.0001; ICGC, *P* = 0.0036; Fudan, *P* = 0.029). Furthermore, we found that our signature remained significant in patients with different histologic grade, TNM stage, locations of tumor entity, age and gender. Multivariate cox regression analysis showed that 6-mRNA signature can be an independent prognostic marker in each of the cohorts. Receiver operating characteristic curve (ROC) analysis also showed that our signature possessed a better predictive role of PDAC prognosis. Moreover, the gene set enrichment analysis (GSEA) analysis showed that several tumorigenesis and metastasis related pathways were indeed associated with higher scores of risk. In conclusion, identifying the 6-mRNA signature could provide a valuable classification method to evaluate clinical prognosis and facilitate personalized treatment for PDAC patients. New therapeutic targets may be developed upon the functional analysis of the classifier genes and their related pathways.

## Introduction

Pancreatic ductal adenocarcinoma (PDAC) stands for more than 90% of cancer cases of the pancreas. It currently ranks as the third common cause of cancer mortality and is predicted to raise to the second by the year 2030. While 5 year overall survival is still lower than 10% and over 50% cases are initially diagnosed in advanced stage where surgical removal is impossible 1, 2, still no standard screening programs are available for high risk patients of pancreatic cancer (like patients with family history or chronic pancreatitis) 3. Currently, the only potentially curative treatment is surgical resection, while adjuvant therapies like chemotherapy with gemcitabine or S-1 (an oral fluoropyrimidine derivative) are usually provided after surgical approaches. As for cases with un-resectable lesions, FOLFIRINOX (fluorouracil, folinic acid [leucovorin], irinotecan, and oxaliplatin) and gemcitabine with nanoparticle albumin-bound paclitaxel (nab-paclitaxel) are the current main therapeutic options. Even targeted molecular therapy (TMT) and immunotherapy are considered in PDAC with a lot of open questions 3. Lacking specific clinical symptoms and biomarkers in early cases and effective therapies based on precise targets are current major challenges in PDAC.

Both tumor tissue biopsy and liquid biopsy samples were applied in exploring potential diagnostic and prognostic markers via high through put technologies such as next generation sequencing on genomics, transcriptomics and proteomics. TCGA: The Cancer Genome Atlas, GEO: Gene Expression Omnibus, and ICGC: International Cancer Genome Consortium are some of the comprehensive database that provide massive array or sequencing-based data for researchers worldwide 4. More and more powerful bioinformatic methods were applied for high through put data analysis. Recently, the cancer Genome Altas Research Network updated an integrated genomic, transcriptomic and proteomic profiling from PDAC samples (including macrodissection or laser capture micro-dissected FFPE for low neoplastic cellularity), which included KRAS mutational heterogeneity and other drivers in wild type tumors, proteomic subtypes with prognostic and therapeutic implications and classification based on mRNA with non-coding RNAs 5. This work is a further development of PDAC molecular signature based on these databases.

Recently, Sharma and colleagues reported that an estimation of pre-diagnostic duration and progression of pancreatic cancer may be provided by fasting blood Glucose levels, indicating that hyperglycemic patients with a mean period of 36 to 30 months before PDAC diagnosis 6. In a different study, Mellby LD et al. developed a liquid biopsy (29 serum biomarkers signature-based) that helps in diagnosing early pancreatic cancer cases in a big Scandinavian PDAC cohort from I to IV stage as compared to 888 control samples and further validated in an independent case-control cohort from United states, the AUC has been achieved to 0.96 7. In addition, a non-invasive three-gene panel was developed from Urine for detecting pancreatic adenocarcinoma as early as possible, while LYVE-1, REG1A and TFF1 were distinguished from healthy controls with a 0.89 by Receiver-operating characteristic curve (ROC) analysis 8. In addition, for the update of PDAC prognostic markers, increasing studies with promising results have been proposed. Shi and the colleagues optimized a 16 mRNA signature from 10 GEO database as an independent prognostic biomarker for recurrence of PDAC patients 9. However, for a practical clinical application of prognostic biomarkers, it is necessary to optimize the signature with the consideration of cost and time.

The aim of this study was identifying potential robust mRNA based signature with minimum amount of genes that may provide a potential prognostic value to develop a fast detection kit based on multiplex PCR method. Therefore, based on previous studies of PDAC biomarkers, we integrated the database from GEO, ArrayExpress, TCGA and ICGC cohort for a comprehensive molecular signature identification and applied the least absolute shrinkage and selection operator (Lasso) regression model to build an optimized and shrunken signature with only 6 genes from mRNA level for predicting both the overall survival and the recurrence of cancer.

## Methods

### PDAC gene expression data

Gene expression profiles or RNA sequencing data of PDACs were downloaded from public GEO (https://www.ncbi.nlm.nih.gov/geo/), Array Express (https://www.ebi.ac.uk/arrayexpress/), TCGA (http://cancergenome.nih.gov/) and ICGC (https://icgc.org/). We retrieved data with respective GSE identification numbers by using “pancreatic” in title in the GEO Repository Browser or ArrayExpress database. We then exported all searching results and chose homo sapiens species datasets. After a careful review of related summaries, five GEO and one ArrayExpress human gene expression datasets were chosen. Exclusion criteria included: un-analyzable datasets; small number of DEGs (less than 100); or incomplete annotated genes (less than 90% of total transcriptomes genes (n< 18000)). For 181 PDAC patients, TCGA database was used to obtain gene expression profile (level 3 data) from RNA-seq and relevant clinical and survival information for Lasso regression. In addition, we also used the 251 cases of GEO samples (including 30 ArrayExpress samples) and 96 cases of ICGC samples with survival for a validation. In addition, we consecutively collected 35 fresh frozen primary PDAC samples at Minhang Hospital from February 2011 to February 2016. Written informed consent were obtained from all patients. The study was conducted in accordance with the Declaration of Helsinki, and the Ethical Committee of Minhang Hospital, Fudan University approved the study.

### The analysis of PDAC gene expression

Data processing was required before the analysis. After downloading and normalizing the raw data CEL files from GEO and ArrayExpress by a robust multiarray averaging (RMA) method 10, we used 'Affy' and 'affycoretools' packages of R software (version 3.3.1, R Foundation for Statistical Computing Vienna, Austria) and processed Affymetrix data. For ICGC and TCGA, available RNA-Seq data were used and log2-transformed.

### Prognostic gene signature identification and validation

'limma' package of R software (version 3.3.1) was used for establishing the prognostic gene signature by generating the differentially expressed mRNAs with* P*-value less than 0.001 and fold-change values of -2 to 2 between tumor and non-tumor samples in GSE15471, GSE28735 and GSE62452 datasets. We then used TCGA dataset and separated prognostic gene signature from other differentially expressed mRNAs.

We then carried the LASSO Cox regression model analysis in the test series of TCGA by using R software (version 3.3.1) and the 'glmnet' package (R Foundation for Statistical Computing, Vienna, Austria). Achieving shrinkage and variable selection simultaneously was guaranteed by using penalized Cox regression model with LASSO penalty, we determined the optimal values of the penalty parameter lambda through 10-times cross-validations 11, 12. Considering the optimal lambda value, we used the gene expression profiling and overall survival (OS) data to screen out a series of prognostic genes and related coefficients from prognostic mRNAs. Prognostic mRNA expression levels of and related coefficient were used to calculate the risk score in each case. Besides, 'Kaps' package was used to find the optimal cut-off value to split the cases into a low / high risk groups. Finally, the Kaplan-Meier and log-rank test assessed the differences in OS and disease free survival (DFS) between the two groups. To validate the results, we used COMBAT (empirical Bayes) to gather samples of GEO and ArrayExpress datasets before analysis, then included them in the inSilicoMerging R/Bioconductor package, as a batch effects removal method. The outcome set which was named as GEO batch series included 251 tumor samples. In addition, ICGC data and Fudan validation series were also used to validate the prognostic signature. A formula similar to test series was used to calculate risk scores and datasets were split into two groups of risks considering the optimal risk score, OS differences were analyzed as mentioned earlier.

### RNA extraction and quantitative reverse transcription PCR (qRT-PCR)

The total RNA of 35 PDAC samples (Fudan validation series) was extracted by using TRIzol reagent (Invitrogen, USA). PrimeScript RT reagent kit (Takara, Japan) was used to carry out reverse-transcription. An ABI Prism 7500 Sequence Detection 0System (Applied Biosystems, Foster City, CA, USA) was applied to do qRT-PCR through using SYBR^®^ Premix ExTaq™ (Takara, Japan). We used GAPDH as an internal control to normalize the expression of mRNAs. The -ΔCT method (ΔCT=CT mRNA - CT GAPDH) was used to calculate mRNA expression levels. The primers of related mRNAs were shown in Table S1.

### Statistical analysis

Cox regression (univariable and multivariable) was carried to study if the prognostic gene signature was affected by age, gender, histological grade, or TNM stage. Associations between high or low-risk groups and clinical pathological aspects were considered using the Student's t or the Fisher's exact test when appropriate. To test whether the risk score can effectively distinguish the two groups of patients, data stratification analysis was performed in the combined datasets by Kaplan-Meier's and the log-rank test according to histological grade, TNM stage, tumor subdivision of pancreas, age and gender. We based the evaluation of specificity and sensitivity of survival prediction on the multi-mRNA risk score, age, histological grade, TNM stage, combined model of risk score and other factors by using Receiver operating characteristic (ROC) analysis. The area under ROC curve (AUC) was considered as an accuracy measure in diagnostic tests 13. The 'pROC' package was adopted for ROC analysis, and the 'delong' method was used to investigate the ROC curves significant differences. OS was identified as the period of time between the diagnosis date and the death date from pancreatic cancer or the date on which data were taken. DFS was calculated from the time of surgery to the time of recurrence or the date on which data were taken. *P* < 0.05 was considered significant for all the Cox regression, log-rank tests and ROC analyses.

### Gene set enrichment analysis

JAVA program (http://www.broadinstitute.org/gsea) was used to complete Gene set enrichment analysis (GSEA) by the use of MSigDB C2 CP: Canonical pathways gene set collection. To make sure that members of a given gene set were commonly related to system of risk score, the GSEA, visualized in Cytoscape (2.8.0), and the Enrichment Map software were utilized 14. Therefore, all mRNA genes in TCGA datasets were ranked with enrichment scores between most positive and most negative. With 1000 random samples per mutations performed, we set the significance threshold at FDR < 0.25 when a gene set had positive score, the majority of included members had higher expression levels and risk score, and the set was labeled “enriched”.

## Results

### Patient Population

The study flowchart was presented in Figure S1. We retrospectively collected eight public mRNA expression datasets of 719 PDAC patients, including 571 tumor tissues and 148 non-tumor tissues. All eligible datasets were summarized in Table S2. Three databases GSE15471, GSE28735 and GSE62452 via affymetrix microarray platform were applied in differentially expressed genes (DEG) analysis 15-17. After the removal of samples with absent clinical outcome information, 528 PDAC patients with survival data were chosen from datasets (Table 1). These included 181 patients from TCGA (test series), 96 patients from ICGC (validation series) and 251 patients from GEO or ArrayExpress (GSE28735, GSE62452, GSE57495, GSE79668 and E-MEXP-2780) as the GEO batch validation series. Table 1 shows that patients ˃ 60y accounted for the vast majority (67.07%), with 53.63% of patients being males. Most cases (80.83%) of the tumor were located in the head of pancreas, histologic grade 2 (55.06%), TNM stage I or II (91.81%). In addition, Table S3 shows the clinicopathological characteristics 35 patients from Fudan validation series.

### Identification of differentially expressed genes

**Figure 1:**
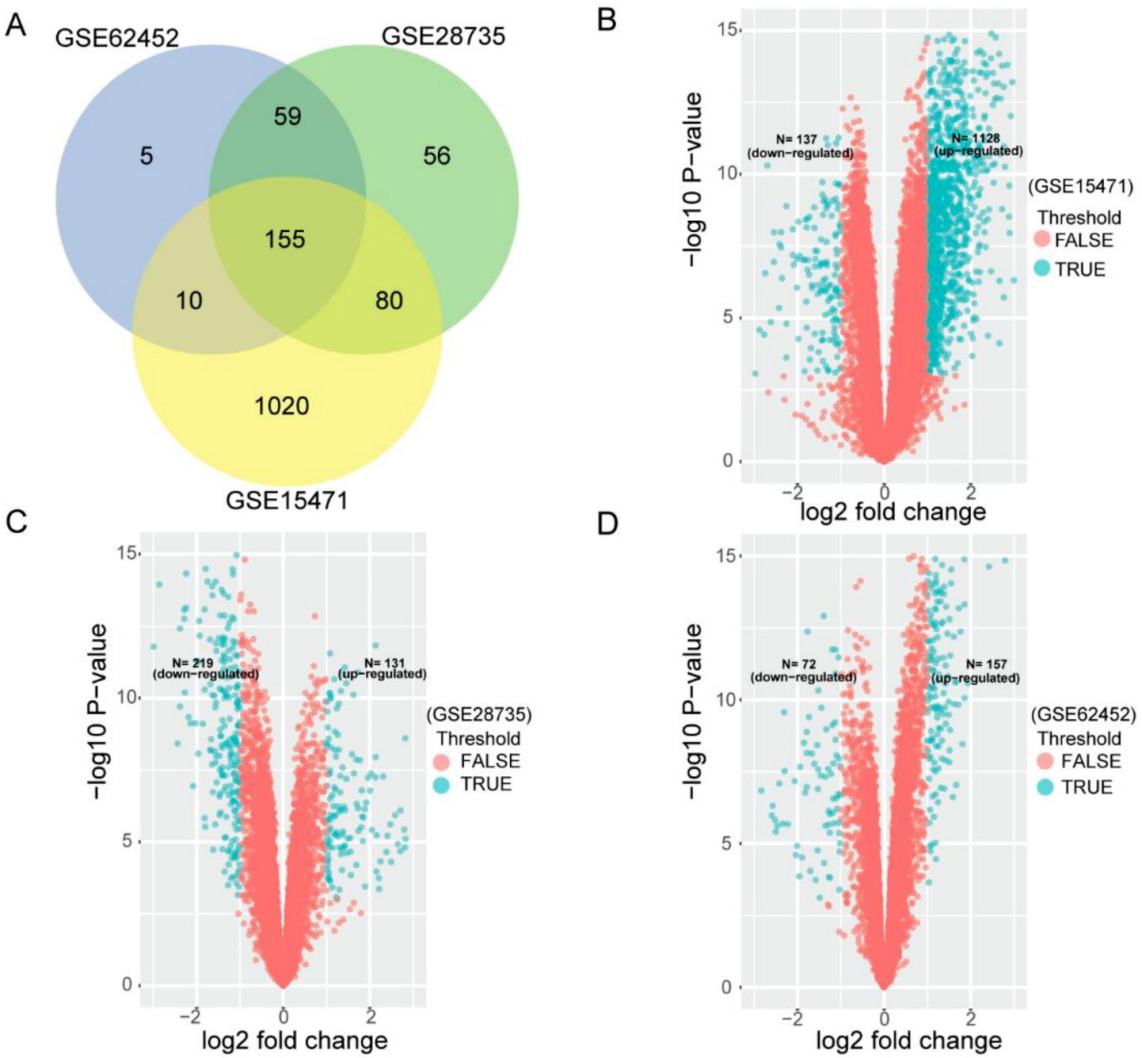
Differentially expressing analyses of genes in GEO datasets. (A) Identification of 155 commonly changed differentially expressed genes (DEGs) from three cohort profile datasets (GSE15471, GSE28735 and GSE62452). Different color areas represent different datasets. The cross areas showed the commonly changed DEGs. DEGs were identified with classical t test; statistically significant DEGs were defined with *P*< 0.001 and |log2 fold change|> 1 as the cut-off criteria. (B) Volcano plots of the DEGs in GSE15471. Among 1265 DEGs, 1128 were up-regulated and 137 were down-regulated. (C) Volcano plots of the DEGs in GSE28735. Among 350 DEGs, 131 were up-regulated and 219 were down-regulated. (D) Volcano plots of the DEGs in GSE62452. Among 229 DEGs, 157 were up-regulated and 72 were down-regulated.

To search for PDAC OS related gene signature, we first used three cohorts (GSE15471, GSE28735 and GSE62452) to analyze DEGs between tumor tissues and non-tumor tissues. After that, we analyzed any overlapping of DEGs. In each venn diagram in all three series, overlapping DEGs were found to be credible. As Figure 1A shows, 155 overlapping differentially expressed mRNAs were identified between the two groups from three cohorts. Figure 1B-D shows datasets' volcano plots of DEGs. In GSE15471, 1128 upregulated and 137 down regulated genes were identified with statistical significance while in GSE28735, we screened only 131 significantly upregulated targets and 219 downregulated targets. In GSE62452 database, among 229 enriched dysregulated genes, there were 157 upregulated and only 72 downregulated genes further identified.

### Establishment of the mRNA prognostic signature

Using the 155 DEGs, we searched for the most discriminating subset of genes that correlated with clinical outcome. To analyze these 155 genes, we applied LASSO Cox regression model in 181 cases from TCGA test series (Figure S2). With this method, we identified a 6-mRNA signature which can predict OS in PDAC cases (Table 2).

**Figure 2:**
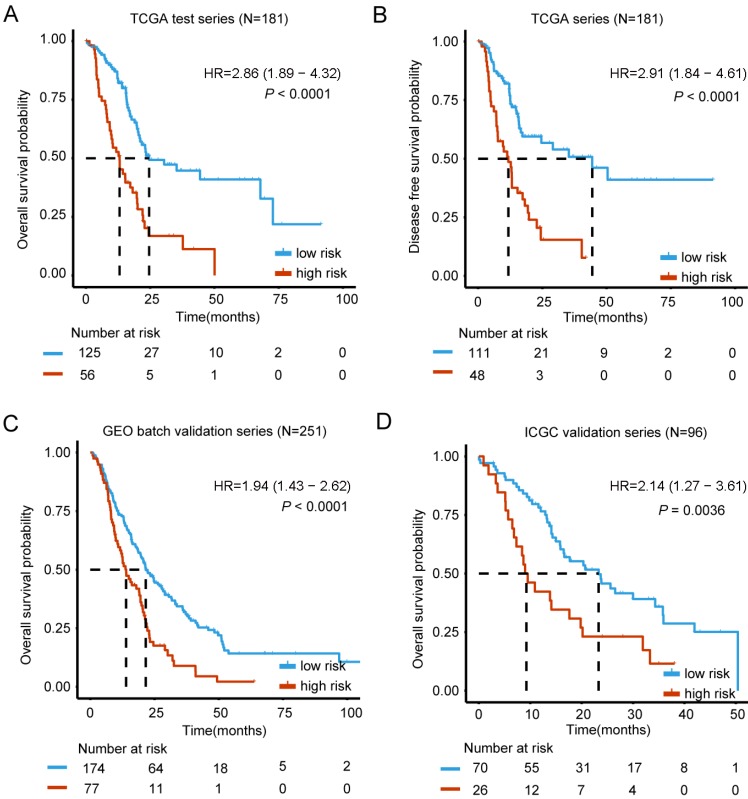
Kaplan-Meier estimates of the overall survival (OS) or disease free survival (DFS) using the six-mRNA signature. The Kaplan-Meier plots were used to visualize the OS or DFS probabilities for the low-risk versus high-risk group of patients based on the best cut-off points (0.1868) of risk score from corresponding TCGA, GEO, ArrayExpress or ICGC datasets. (A) Kaplan-Meier curves for OS in TCGA test series patients (N= 181); (B) Kaplan-Meier curves for DFS in TCGA series patients (N= 181) (C) Kaplan-Meier curves for OS in GEO batch validation series patients (N= 251); (D) Kaplan-Meier curves for OS in ICGC validation series patients (N= 96). The tick marks on the Kaplan-Meier curves represent the censored subjects. The differences between the two curves were determined by the two-side log-rank test. The number of patients at risk was listed below the survival curves.

Higher levels of genes expression were indicated by positive coefficients. Interestingly, all of the 6 genes had positive coefficients - Kynureninase (KYNU), Hepatocyte growth factor receptor-MET Proto-oncogene (MET), Inositol Polyphosphate-4-Phosphatase Type IIB (INPP4B), Insulin Like growth Factor 2 mRNA binding protein 3 (IGF2BP3), Ankyrin Repeat Domain 22 (ANKRD22) and DNA Topoisomerase II Alpha(TOP2A). We based the calculation of each case's risk score on the 6 genes' expression levels and related coefficients in the multivariate model (Table 2). Risk score = (0.067744*expression level of KYNU) + (0.332037*expression level of MET) + (0.012583*expression level of INPP4B) + (0.003424* expression level of IGF2BP3) + (0.012010* expression level of ANKRD22) + (0.041295* expression level of TOP2A). As expected, the classifier based on this 6-mRNA signature divides all cases into a high risk (N=56) and a low risk group (N= 125), which can most significantly distinguish the outcome of these PDAC patients (Figure 2A). Notably, only up to 1.8% patients of the high risk group survived beyond 50 months compared to 8% of the low risk group. In addition, high and low-risk group displayed clear statistical difference in both OS and DFS curve (*P* < 0.0001, Figure 2A-B).

Recently, a number of PDAC prognostic gene signatures have been found to display robust and independent prognostic value 18-20. We evaluated any genetic overlapping among these signatures and our 6-mRNA signature. As shown in Figure S3, there was only one gene (IGF2BP3) in common between our 6-mRNA signature and Chen *et al*., and the overlap of the signature was very low.

### Validation of the 6-mRNA prognostic signature and clinicopathological associations

For results confirmation, the validation of our 6-mRNA signature was carried in two independent datasets (GEO batch series and ICGC series). By using 6-mRNA signature classifier, cases were split into high / low risk groups in the validation series. Consistent with the previous findings, high risk group in GEO batch series showed a significantly shorter median OS compared to the other group (HR = 1.94, 95% CI 1.43-2.62, *P* < 0.0001) (Figure 2C). Importantly, analysis in the ICGC and Fudan validation series also showed similar results (HR = 2.14, 95% CI 1.27-3.61, *P* = 0.0036; HR = 3.11, 95% CI 1.07-9.05, *P* = 0.029) (Figure 2D, Figure S4), thus it confirms the prognostic value. Interestingly, Chi-square test was also implemented to study whether the 2-year OS is associated with risk classification (low vs. high risk group), and the difference was significant in TCGA, GEO batch and ICGC series (Table S4). In conclusion, 6-mRNA prognostic signature represents an optimal combination of highly reproducible and robust prognostic genes.

In addition, we investigated associations between the 6-mRNA signature-based classification (in the two groups) and clinicopathological variables of samples in TCGA, GEO batch, ICGC and Fudan validation series. As Table S4 shows, no significance was found regarding patients' age, gender, tumor location of Pancreas or TNM stage. Conversely, histologic grade was found to be significant between the low and high risk groups in TCGA test series and GEO batch series (*P* = 0.043, *P* = 0.004; respectively).

### Univariate and multivariate analyses of the 6-mRNA signature prognostic abilities

**Figure 3:**
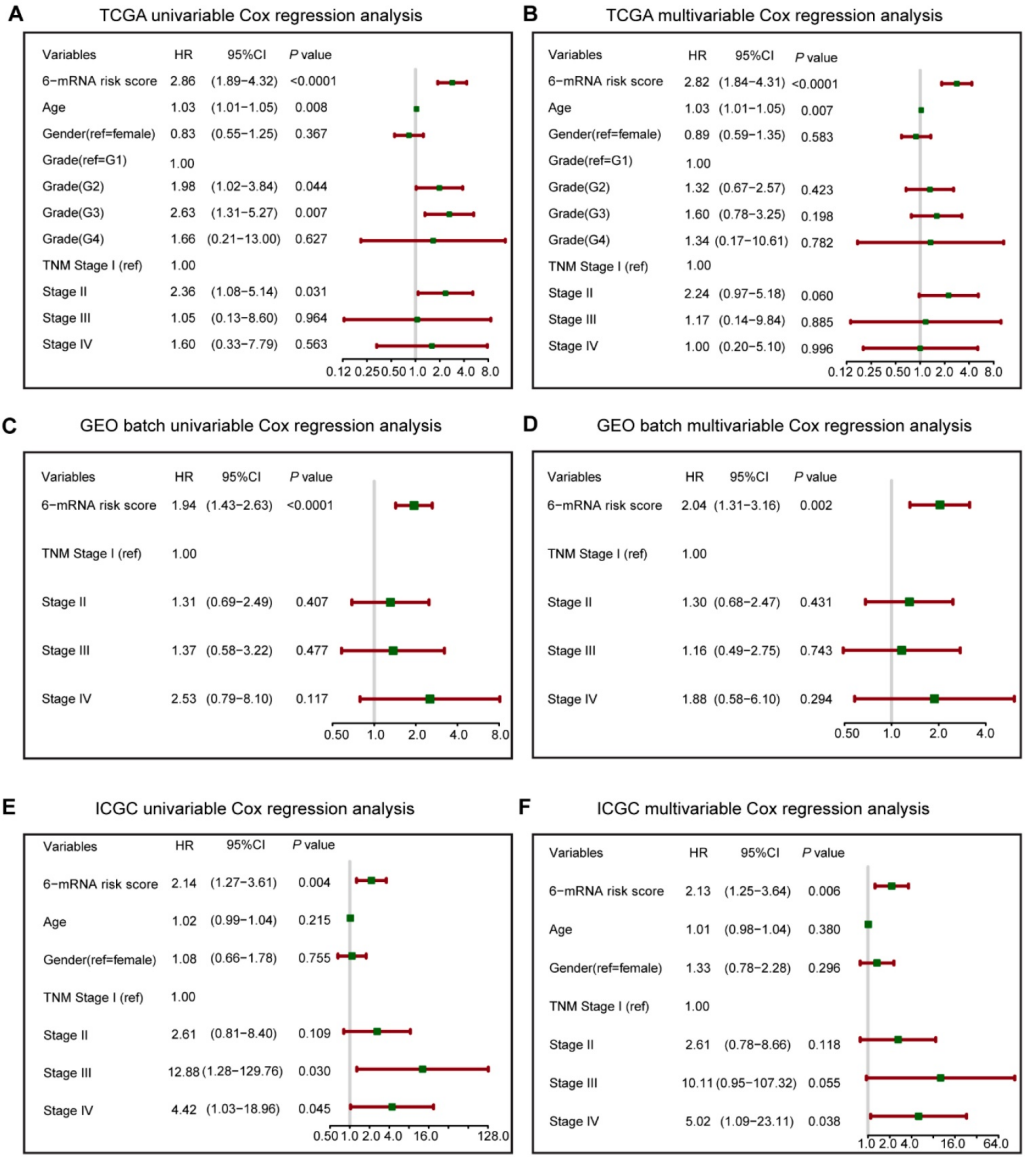
Forest plot summary of analyses of overall survival (OS). Univariable and multivariable analyses of the six-mRNA risk score, age, gender, histological grade and TNM stage on TCGA (A, B), GEO batch (C, D) and ICGC datasets (E, F). The green squares on the transverse lines represent the hazard ratio (HR), and the red transverse lines represent 95% CI. Risk score and age are continuous variables, gender, histological grade and TNM stage are discontinuous variables.

To verify the prognostic value of 6-mRNA signature independently from the clinicopathological characteristics, we performed COX univariable and multivariable analysis that included 6-mRNA risk score, age, gender, histologic grade and TNM stage (When available) as co-variables in three datasets. In univariate analysis (Figure 3), three variables were found to be associated with OS, including the Grade, Age, TNM stage, and our 6-mRNA signature. The multivariate modelling results further supported the contention that the 6-mRNA risk score was indeed associated with OS even after considering other clinical factors in each dataset (Figure 3A-F) and combined datasets (Figure S5A-B). Additionally, the univariate and multivariate analyses in Fudan validation series also had the similar results (Figure S6A-B). Therefore, the prognostic values of 6-mRNA signature are independent from all other clinical variables tested.

### The stratification analysis based on clinicopathological characteristics

**Figure 4:**
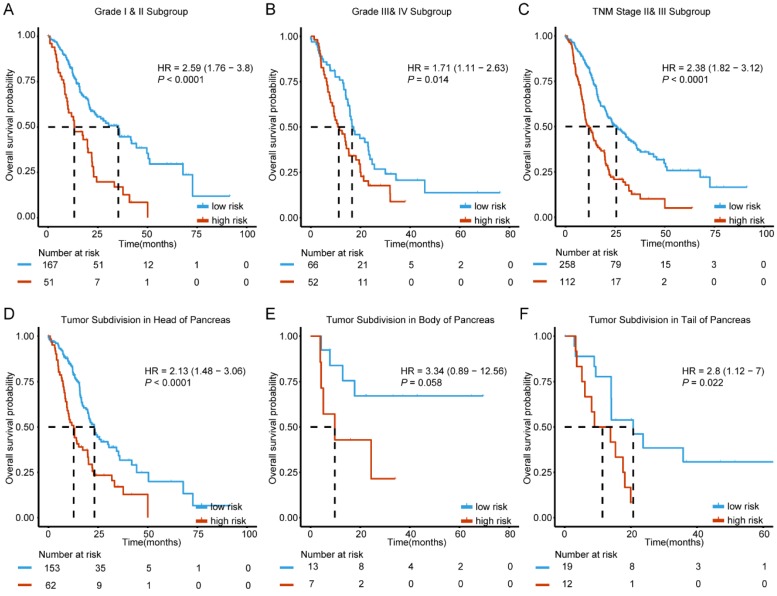
Kaplan-Meier survival analysis to evaluate the independence of the six-mRNA signature from histological grade, TNM stage and tumor subdivision of pancreas. The patients from combined datasets were stratified into subgroups. The six-mRNA signature was applied to the low histological grade patients (A), high histological grade patients (B), TNM stage II and III patients (C), patients with tumor subdivision in head (D), body (E) and tail (F) of pancreas, separately. The number of patients at risk was listed below the survival curves. The tick marks on the Kaplan-Meier curves represent the censored subjects. The differences between the two curves were determined by the two-sided log-rank test.

Since clinicopathological characteristics may also possess prognostic value, we evaluated the predictive ability of our molecular signature in different clinical factors. Based on histologic grade, cases were split into two sub-groups with histologic grade I and II defined as well differentiated stratum and grade III and IV as poor differentiated stratum. The stratification analysis indicated that the 6-mRNA signature could help in identifying differences in prognosis among patients in the two tumor grade subgroups. (Figure 4A-B). In addition, further analysis by stratification of the TNM staging variable showed that the 6-mRNA signature could also divide cases into low and high risk in each stage with a statistically significant difference at stage II&III (HR=2.38, 95% CI: 1.82-3.12, *P* < 0.0001) (Figure 4C). The results indicated that the 6-mRNA signature prognostic value is independent from histologic grade and tumor stage.

According to locations of the tumor entity for PDAC, patients in all combined series were split between three subgroups (Head, Body, and Tail). The stratification analysis showed that 6-mRNA signature may indeed help in identifying cases with prognostic differences in each subgroup (HR=2.13, 95%CI: 1.48-3.06, *P* < 0.0001; HR=2.8, 95% CI: 1.12-7.0, *P* = 0.022, respectively) despite the absence of statistical significance in the tumor subdivision in the body of pancreas (Figure 4D-F). This might be due to the small sample size (20 patients only, Figure 4E). However, 6-mRNA signature still showed a tendency to distinguish cases with different prognoses.

Aging is one of the factors affecting cancer progression 21. Data stratification analysis was carried which split these cases into ≤ 60y and ˃ 60y subgroups. After age stratification, 6-mRNA based signature remained powerful for predicting OS. In patients from the two different age groups, the 6-mRNA signature could identify cases with different prognosis (Figure S7A-B). Regarding the gender of PDAC patients, 6-mRNA risk score also had the ability to predict the OS in either female or male population within the combined series (HR=1.92, 95% CI: 1.28-2.88, *P* = 0.0013; HR=2.69, 95% CI: 1.85-3.92, *P* < 0.0001; Figure S7C-D). The results indicated that 6-mRNA signature prognostic value is indeed independent from age and gender.

### ROC analysis for the comparison between the models with other prognostic factors

**Figure 5:**
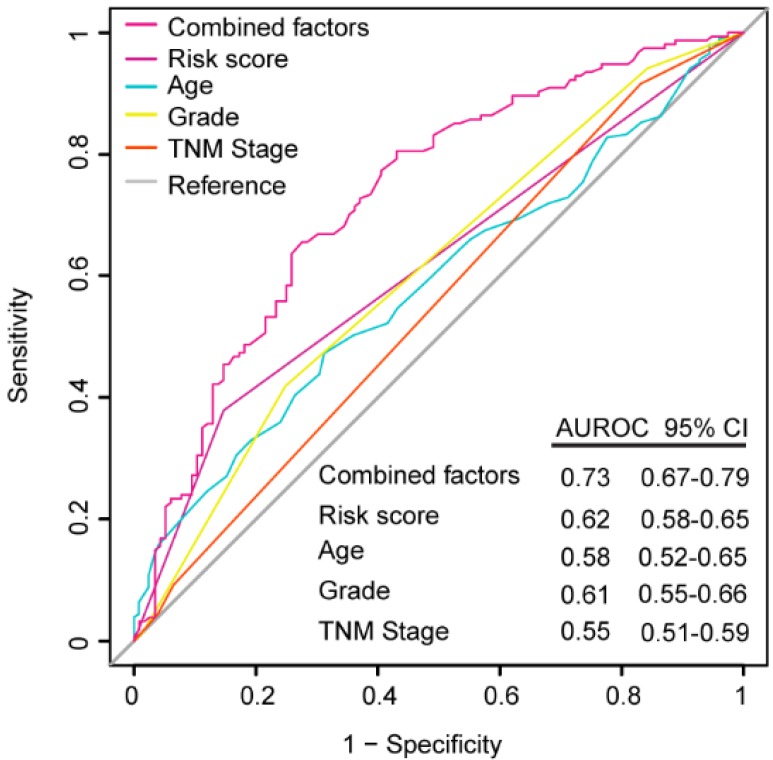
Receiver operating characteristic (ROC) analysis of the sensitivity and specificity of the overall survival (OS) prediction by the six-mRNA risk score, age, histological grade, TNM stage and all combined factors in combined datasets patients (N= 528). *P* values were from the comparisons of the area under the ROC (AUROC) of all combined factors versus six-mRNA risk score, age, histological grade and TNM stage, respectively. As can be seen, the six-mRNA risk score combined with other factors showed a better prediction of OS than age (*P* < 0.0001), histological grade (*P* < 0.0001) and TNM stage (*P* < 0.0001).

ROC analysis was performed to evaluate if the 6-mRNA signature could improve the clinicopathological characteristics for predicting better prognosis and demonstrate the survival prediction specificity and sensitivity with and without other parameters combination. As Figure 5 shows, the 6-mRNA risk score possessed a better predictive role of PDAC prognosis than age, histology grade and TNM stage in combined datasets patients. What's more, when 6-mRNA risk score was combined with age, grade and tumor stage, significant differences were found among the combined factors and the 6-mRNA risk score (0.73 versus 0.62, 95% CI 0.67-0.79 versus 0.58-0.65, *P* < 0.0001). That could indicate a stronger role of 6-mRNA signature in OS prediction when combined with other prognostic factors in ROC analysis.

### Identification of 6-mRNA signature related biological pathways / processes

**Figure 6:**
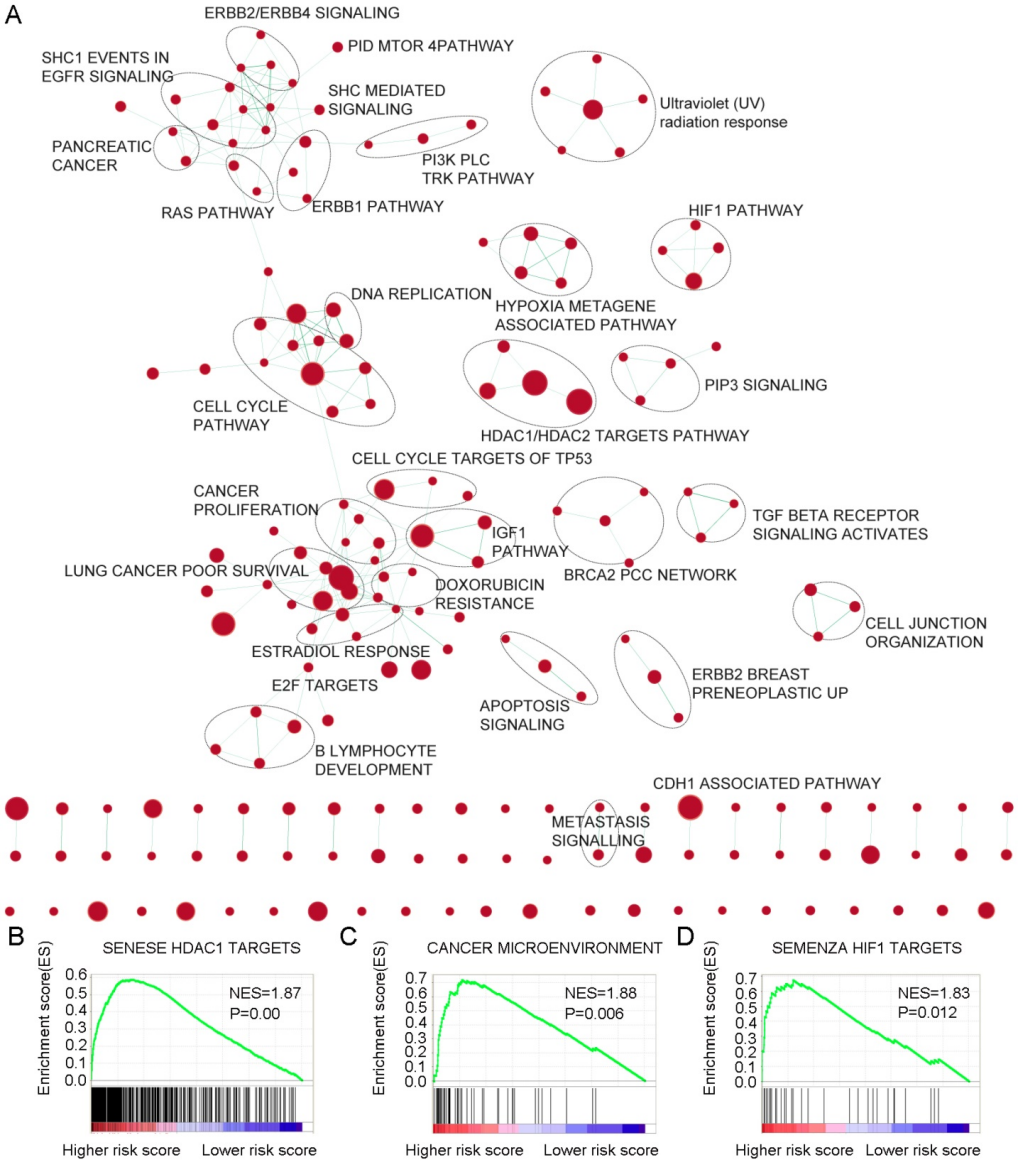
Gene Set Enrichment Analysis Delineates Biological Pathways and Processes associated with risk score. Cytoscape and Enrichment Map were used for visualization of the GSEA results. Nodes represent enriched gene sets, which are grouped and annotated by their similarity according to related gene sets. Enrichment results were mapped as a network of gene sets (nodes). Node size was proportional to the total number of genes within each gene set. Proportion of shared genes between gene sets was represented as the thickness of the green line between nodes.

For the identification of possible related biological process and signalling pathways, GSEA was performed by using 6-mRNA signature based on risk scores for prognostic classification. Several cancer-related networks were found to be up regulated in high risk score cases including cell cycle/DNA replication, cancer microenvironment, Hypoxia metagene associated pathways/HIF1 pathway and some therapeutic targets correlated signalling such as RAS, ERBB1/2/4, PI3K PLC TRK, IGF1, mTOR, TGF-b receptor, HDAC1/2 targets, and CDH1 associated pathways (Figure 6A-D, Table S5). For instance, HDAC (histone deacetylase) enzymes which play an essential role in cancer development and progression and HDACIs (HDAC inhibitors) have been found to affect differentiation and cell cycle arrest, activate the apoptosis related extrinsic / intrinsic pathways, prevent metastasis and angiogenesis, and restore the chemotherapy sensitivity in different cancer cell lines including pancreatic cancer. More preclinical evidences and clinical trials are necessary to determine the efficiency of these novel epigenetic modulation associated therapies 22, 23. We suggested that the 6-mRNA signature may indeed be involved in such networks.

## Discussion

The biggest challenges of pancreatic cancer are the early diagnosis, precise prediction of tumor progression and the intervention of the late stage of the disease. Promising biomarkers and advanced imaging techniques as well as interdisciplinary treatments of this disease will help to develop precision medical strategies in pancreatic cancer.

In our study, we found a novel mRNA signature that can serve as a prognostic biomarker for overall survival of the disease. To obtain 155 DEGs, we used a strategical stepwise analysis to spot any possible overlapping. Afterwards, LASSO COX regression model was constructed based on these genes. Ten fold cross validation was applied to choose the optimal choice with minimum error of mean cross validation from a models' sequence. In addition, LASSO coefficient profiles were used while we produced a coefficient profile plot against the Log Lambda sequence and optimal Lambda that resulted in 6 non-zero coefficients. All 6 genes enriched in our signature were identified as upregulated in tumor samples. We found that MET has the highest coefficient value, which indicated that the significance of this gene might be involved in PDAC. A number of studies have demonstrated that poor prognosis is in a correlation with abnormal MET activation in cancer, and cancer growth, angiogenesis, and metastasis are triggered by MET activation 24, 25. MET is found to be deregulated in many human cancers, including breast, kidney, stomach, liver, and brain cancer. That led to proposing MET amplification as a potential biomarker for cell tumor 26. MET role in pancreatic cancer has also been highlighted. Cuneo and the team used tissue microarray to show high levels of c-MET expression in tumor was linked to faster distant failure in neoadjuvant therapy patients (median 8.9 months vs 22.0 months, *P* = 0.0010) 27.

Beside the fact that a well known oncogene (MET) was included, some metabolic regulating genes were also enriched. For example, KYNU is an enzyme regulating serum kynurenine metabolites. Higher serum concentrations of (HAA/HK) ratio (3-hydroxyanthranilic acid / HAA: 3-hydroxykynurenine) (PLP in vivo functional measure) was significantly correlated with reduced risk of pancreatic cancer 28. Chen and the team found that the overexpression of INPP4B (a phosphatidylinositol signalling pathways involved enzyme) can suppress the invasion, migration, and angiogenesis of prostate cancer, they also were able to show INPP4B reversed docetaxel resistance and EMT (epithelial to mesenchymal transition) through PI3K/Akt pathway 29, 30. Kofuji's work further discovered that in PTEN deficiency, INPP4B acts as tumor suppressor with very important functions 31. Our findings indicated a future interest of this gene modulation in pancreatic cancer progression. IGF2BP3 has been also reported as biomarker with clinical relevance in different type of cancers including Glioma, colorectal, lung, liver, and breast cancer 32; accumulating evidence also demonstrated this gene role in pancreatic cancer with the perspectives of migration, invasion, and adhesion as well as IGF2BP3-mediated translation in cell protrusions 33, 34. It is very interesting to get some hints of this signature in DNA metabolism, since TOP2A was screened in our analysis. TOP2A acts as a target for many anti-cancer agents, with many mutations in it being associated with drug resistance. Several new studies updated its prognostic role as a powerful therapeutic target in pancreatic cancer 35, 36. This may also indicate a potential correlation of this signature with PDAC chemotherapy response.

In our study, we proved that 6-mRNA signature is an independent powerful factor for PDAC patients OS prognosis. Compared to other known signatures to predict OS 18-20, our model was obtained from more comprehensive databases and it seemed to be easier to manipulate in practical clinical application with minimum amount of genes. Furthermore, Raman et al used the similar database to generate a 5-gene signature to predict the outcome of PDAC patients, but they adopted iterative sampling based algorithms 37. In addition, to our knowledge, Shi et al established a 16-mRNA based signature at a minimum lambda with minimum error of mean cross validation via LASSO model in PDAC and could predict RFS for PDAC patients^9^. So we also tried our best to test whether our 6-mRNA signature can predict DFS for PDAC patients in TCGA series which survival data can be acquired, and we found that our signature had the ability to predict in both overall and disease free survival curves.

Many studies have proved that tumor grade was a highly important prognostic factor in PDAC 38, 39. Compared to other PDAC molecular signatures, our 6-mRNA signature was able to predict the OS in both well and poor differentiated cases. In addition, our data also showed that this unique signature was able to predict the survival with in the tumor stage II&III. However, the TNM stage was not an independent marker for prognosis neither in TCGA, ICGC nor GEO batch cohort. Although in combined univariable cox regression analysis, TNM I, II and IV (compared to TNM I) showed statistical significance in hazard ratio, yet only TNM II kept the statistical difference in multivariable cox regression analysis. This might be due to the disproportion of subgroups distribution. Nearly more than 50% patients were in II stage tumors but not high proportion in III and IV stages.

The importance of the location of PDAC as predictor of survival has also been described by many studies 40, 41. Our study is the first to demonstrate that 6-mRNA signature had the ability to predict OS with in different tumor subdivision of pancreas either in head or in the tail. Due to the small size of the population, there was no statistical significance for the tumor in the body of pancreas. However, 6-mRNA signature still showed a tendency to distinguish cases with different prognosis.

Beside, as we know in pancreatic cancer, aging is considered as a high risk factor, most patients are diagnosed in old ages 21. Within our model, both young and old patients could be split into high / low risk for prognosis. However, one consideration was the cut-off value of young and old patients. In a deutch pancreatic cancer group study, they used the age of 75 year-old to group the young and old patients, and found that older age is independently associated with worse OS in non-resected, non-metastatic pancreatic cancer 42. Furthermore, our 6-mRNA signature independently predicted patients' survival (stratifying patients with gender), which suggests that this signature could serve as a powerful prognostic indicator for both female and male patients.

ROC analysis showed that in PDAC prognosis evaluation (in combined datasets), this 6-mRNA signature was superior to age, histology grade and TNM stage. Many clinical parameters might affect the survival after PDAC surgery like histologic grade, TNM stage, serum CA19-9, perioperative blood transfusion, and metastasis to lymph nodes 43-49. To enhance prognostic prediction, 6-mRNA risk score was combined with age, histologic grade and tumor stage. Significant differences were found between the combined factors and the 6-mRNA risk score, indicating that our 6-mRNA signature combined with other prognostic factors may have a stronger power for OS prediction.

As the 6-mRNA signature was able to distinguish high risk cases based on risk score, the involved molecular mechanisms still need to be studied. The biological behavior of 6-mRNA signature supported the layout of GSEA we produced in this study. A number of important functional pathways were highlighted such as cancer microenvironment, Hypoxia metagene associated pathway, and IGF1 pathway. In addition, HDAC, HIF1, ERBB, E2F, TGFb, and CDH1 were also visualized in the map for therapeutic targets indication. Therefore, significant signalling pathways may indeed support that 6-mRNA signature has OS and DFS prediction ability and provide potential methods for any future targeted therapies.

Nonetheless, there are some limitations in our study. First, preoperative serum CA19-9 level is the only U.S. FDA (Food and Drug Administration) approved biomarker and the most widely used in the U.S. 29, 30. Since it can serve as a diagnostic and prognostic biomarker, we, however, did not acquire and generate the correlation of our mRNA signature genes with this marker or combine these serum markers (such as CA19-9 or CA125) with our risk marker for a combination score to predict the prognosis. Second, our validation datasets (GEO batch and ICGC) consisted only of OS, which cannot further validate our signature effectively. Third, our study is retrospective and large prospective cohorts are needed to validate the utility of this signature in prognostic classification.

Last but not least, as an important integrated analysis of pancreatic cancer mentioned in the introduction part, a precise prediction of PDAC characterization and prognosis via multi-platform analysis combing genomics, transcriptomics, epigenetics and proteomics will be beneficial for a complex molecular landscape of PDAC and provide a road map for precision medicine.

## Conclusions

Our data defined a unique 6-mRNA signature based on comprehensive bioinformatics analysis and associated with prognosis independently of classical prognostic factors. Moreover, this 6-mRNA predictive signature remained significant in patients with different subtypes. Our finding indicated that our 6-mRNA signature may contribute to personalized management of PDAC patients. Of course, future investigations on the prospective and large sample study for the 6-mRNA signature will be of great clinical significance. In addition, with novel therapies being developed, some classifier genes or pathways in which our 6-mRNA signature is involved may represent novel therapeutic targets for PDAC treatment.

## Supplementary Material

Supplementary Figures and Tables.

Supplementary Table S2.

## Figures and Tables

**Table 1:** Patient and tumor clinicopathological characteristics of 528 pancreatic adenocarcinoma patients involved in the study.

Characteristics	All (N=528)	Detailed data		
		TCGA (N=181)	GEO batch (N=251)	ICGC (N=96)
**Age at diagnosis, years**				
≤60	108 (32.93%)	61 (18.60%)	18 (5.49%)	29 (8.84%)
˃60	220 (67.07%)	120 (36.59%)	33 (10.06%)	67 (20.43%)
Gender				
Female	166 (46.37%)	81 (22.63%)	38 (10.61%)	47 (13.13%)
Male	192 (53.63%)	100 (27.93%)	43 (12.01%)	49 (13.69%)
**Tumor location of Pancreas**				
Head	215 (80.83%)	139 (52.26%)	—	76 (28.57%)
Body	20 (7.52%)	15 (5.64%)	—	5 (1.88%)
Tail	31 (11.65%)	16 (6.02%)	—	15 (5.64%)
**Histologic grade**				
G1	33 (9.82%)	30 (8.93%)	2 (0.60%)	1 (0.30%)
G2	185 (55.06%)	97 (28.87%)	32 (9.52%)	56 (16.67%)
G3	113 (33.63%)	50 (14.88%)	29 (8.63%)	34 (10.12%)
G4	5 (1.49%)	2 (0.60%)	1 (0.30%)	2 (0.60%)
**TNM stage**				
I	47 (11.66%)	21 (5.21%)	17 (4.22%)	9 (2.23%)
II	323 (80.15%)	149 (36.97%)	94 (23.33%)	80 (19.85%)
III	16 (3.97%)	4 (0.99%)	11 (2.73%)	1 (0.25%)
IV	17 (4.22%)	5 (1.24%)	6 (1.49%)	6 (1.49%)

TNM, tumor-nodes-metastasis; —: Without available data.

**Table 2:** mRNAs significantly associated with the overall survival in the test series patients (N=181)

Gene symbol	Coefficient	Description
KYNU	0.067744	Kynureninase (L-Kynurenine Hydrolase)
MET	0.332037	MET Proto-Oncogene, Receptor Tyrosine Kinase
INPP4B	0.012583	Inositol Polyphosphate-4-Phosphatase Type II B
IGF2BP3	0.003424	Insulin Like Growth Factor 2 MRNA Binding Protein 3
ANKRD22	0.012010	Ankyrin Repeat Domain 22
TOP2A	0.041295	DNA Topoisomerase II Alpha

## Abbreviations

AUC: Area under the curve
CI: Confidence interval
DEGs: Differentially expressed genes
DFS: Disease free survival
FDR: False discovery rate
GEO: Gene Expression Omnibus databases
GSEA: Gene set enrichment analysis
HR: Hazard ratio
ICGC: International Cancer Genome Consortium
LASSO: least absolute shrinkage and selection operator
Limma: Linear Models for Microarray Data
mRNAs: Messenger RNAs
OS: Overall survival
PDAC: Pancreatic ductal adenocarcinoma
RMA: Robust multiarray averaging
ROC: Receiver operating characteristic curve
TCGA: The Cancer Genome Atlas
