# Supplementary material for: A robust 6-mRNA signature for prognosis prediction of pancreatic ductal adenocarcinoma
Source: Int J Biol Sci. 2019 Aug 22;15(11):2282–95. doi: 10.7150/ijbs.32899 (PMC6775308; doi:10.7150/ijbs.32899)
Supplement: Supplementary file 1 — Supplementary Figures and Tables. [file ijbsv15p2282s1.pdf]

Supplementary materials

A robust 6-mRNA signature for prognosis prediction of pancreatic ductal adenocarcinoma

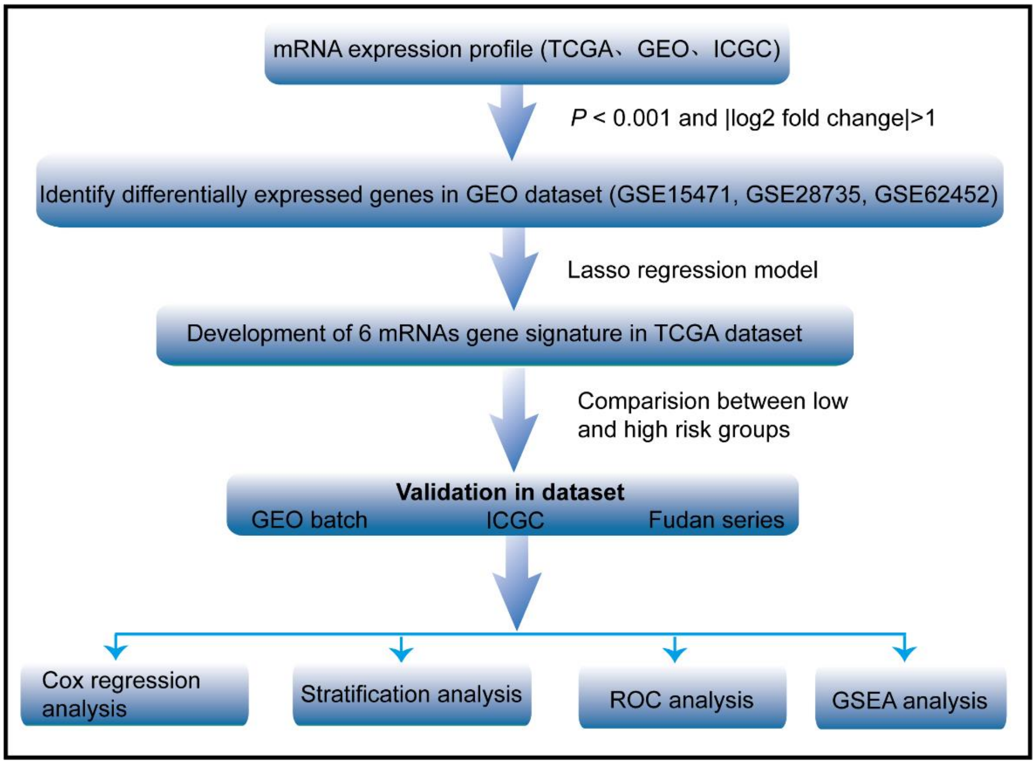

Figure S1 Flow chart of the study. The study was performed in eight public mRNA expression datasets and Fudan PDAC patients. Three database GSE15471, GSE28735 and GSE62452 via affymetrix microarray platform were used to identify differentially expressed genes. The 181 patients from TCGA used to establish a prognosis model based on differentially expressed genes. The prognosis analysis was validated using independent 251 samples from GEO batch, 96 patients from ICGC and 35 patients from Fudan validation series, respectively.

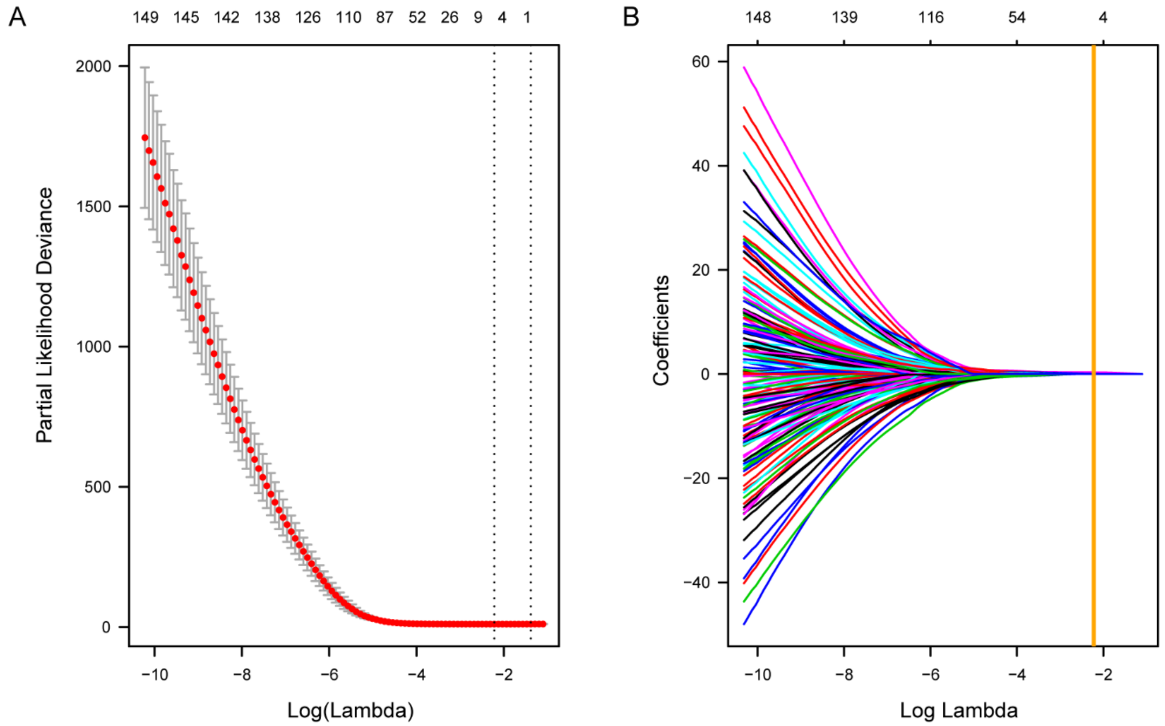

Figure S2 Texture feature selection using the least absolute shrinkage and selection operator (LASSO) cox regression model. (A) Cross-validation for tuning parameter selection in the LASSO model. The solid vertical lines are partial likelihood deviance ± standard error (SE). The dotted vertical lines are drawn at the optimal values by minimum criteria and 1-SE criteria. We plotted the partial likelihood deviance versus Log (Lambda), where Lambda is the tuning parameter. Herein, a value Lambda = 0.1087 with Log (Lambda) = -2.2192 was chosen by 10-time cross validation via 1-SE criteria. (B) LASSO coefficient profiles of the texture features. A coefficient profile plot was produced against the Log Lambda sequence. Vertical lines was drawn at the value selected using 10-fold cross-validation, where optimal Lambda resulted in six nonzero coefficients.

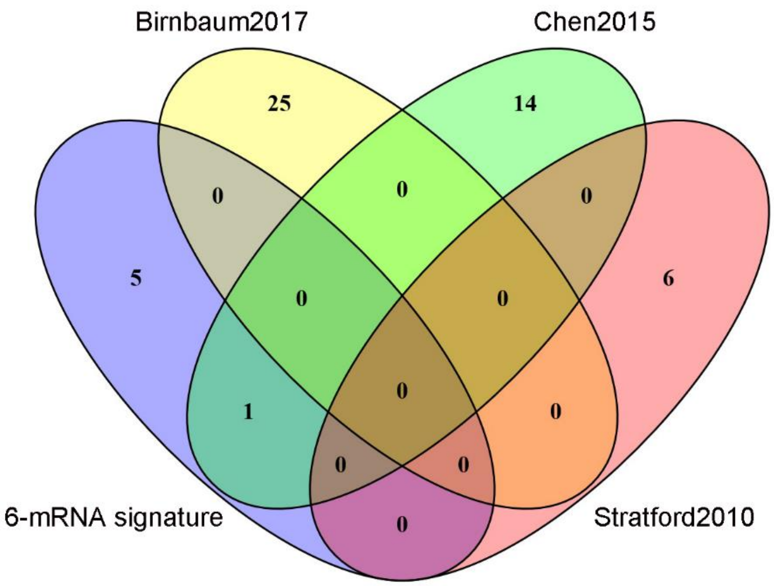

Figure S3 Gene overlap between our 6-mRNA signature and other prognostic signatures. Venn diagram showing the overlap in genes between our signature and three prognostic signatures.

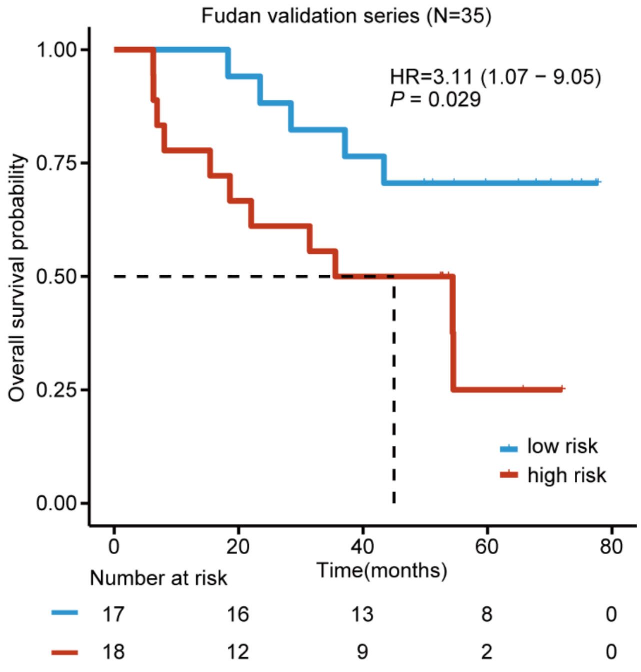

Figure S4 Kaplan-Meier curves for OS in Fudan validation series patients (N= 35).

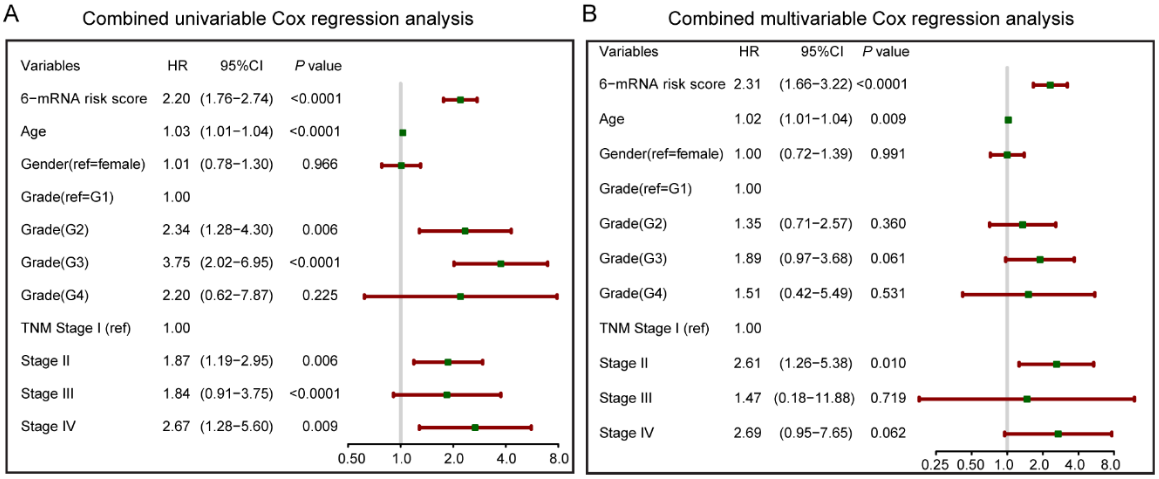

Figure S5 Forest plot summary of analyses of overall survival (OS). Univariable and multivariable analyses of the six-mRNA risk score, age, gender, histological grade and TNM stage on combined datasets (A, B). The green squares on the transverse lines represent the hazard ratio (HR), and the red transverse lines represent 95% CI. Risk score and age are continuous variables, gender, histological grade and TNM stage are discontinuous variables.

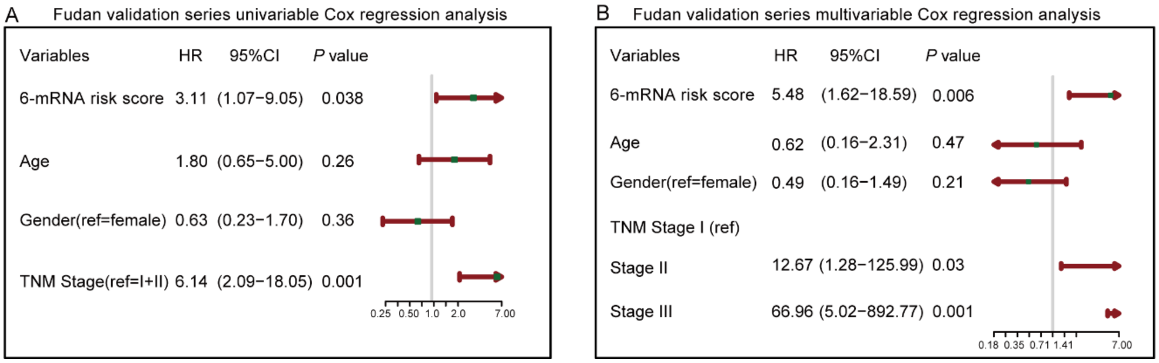

Figure S6 Forest plot summary of analyses of overall survival (OS). Univariable and multivariable analyses of the six-mRNA risk score, age, gender and TNM stage on Fudan validation series (A, B). The green squares on the transverse lines represent the hazard ratio (HR), and the red transverse lines represent 95% CI.

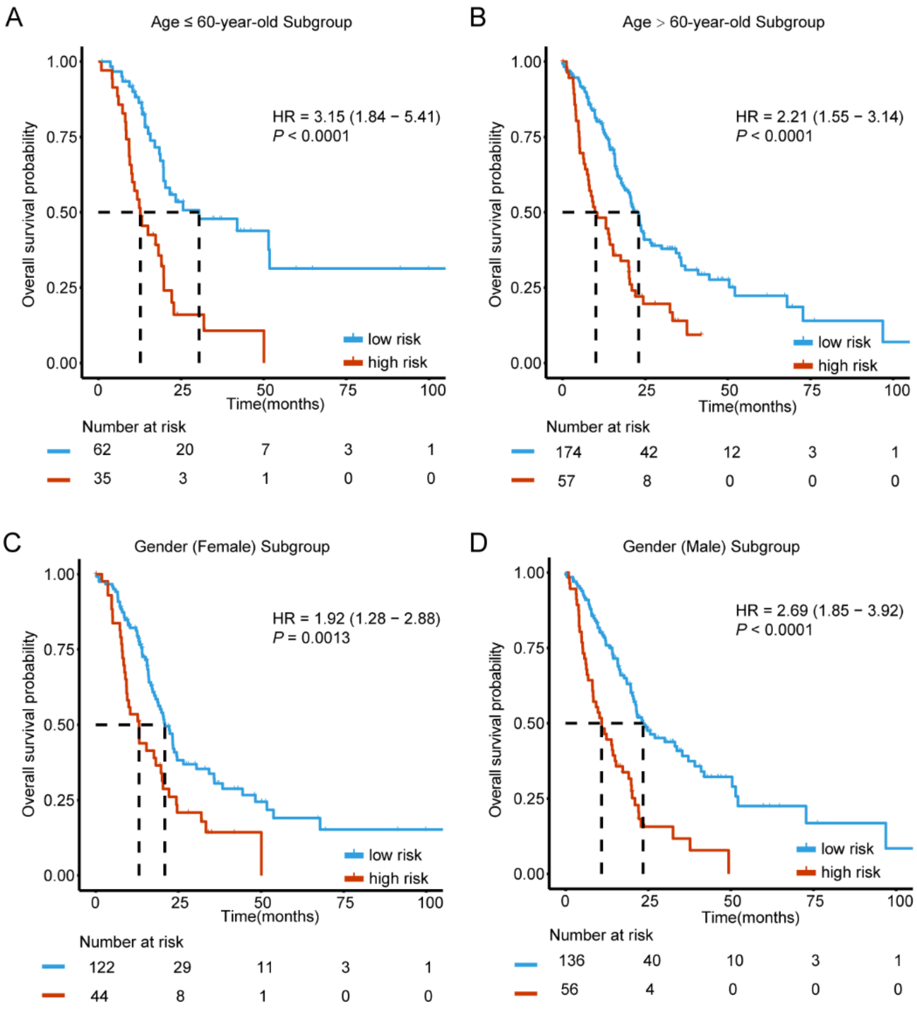

Figure S7 Kaplan–Meier survival analysis to evaluate the independence of the six-mRNA signature from age and gender. The patients from combined datasets were stratified into subgroups. The six-mRNA signature was applied to the age ≤ 60-year-old patients (A), age > 60-year-old patients (B), female patients (C), male patients (D), separately. The number of patients at risk was listed below the survival curves. The tick marks on the Kaplan–Meier curves represent the censored subjects. The differences between the two curves were determined by the two-sided log-rank test.

Table S1. Primers of six mRNAs and internal control for qRT-PCR.

**Primers**		**5'→3'**
KYNU	Forward	TGGCTCCAGTTCCTCTCTATAA
	Reverse	ACCACAGCAGCTTTCAGTATAA
MET	Forward	CTGGACAGATTGTGGGAGTAAG
	Reverse	TTCAGTTCAGCTGCAGGTATAG
INPP4B	Forward	CTGATGCTGACGCTAAGAAGAG
	Reverse	TAGGAAGCCTGGGTCATACA
IGF2BP3	Forward	TACGGAGCAGTGCCTAGTAT
	Reverse	TCCTGCGACCACTTTGTTAG
ANKRD22	Forward	TGCTGGCGTCGAAGTTAAT
	Reverse	CTTCCAAGAGCAGAGGGATAAG
TOP2A	Forward	GACGCTTCGTTATGGGAAGATA
	Reverse	GGGCCAGTTGTGATGGATAA
GAPDH	Forward	CTGGGCTACACTGAGCACC
	Reverse	AAGTGGTCGTTGAGGGCAATG

Table S3. Patient and tumor clinicopathological characteristics of Fudan validation series.

Characteristics	Fudan validation series (N=35)
Age at diagnosis, years	
≤60	17 (48.57%)
>60	18 (51.43%)
Gender	
Female	16 (45.71%)
Male	19 (54.29%)
TNM stage	
I	8 (25.00%)
II	15 (46.88%)
III	9 (28.13%)

TNM, tumor-nodes-metastasis

Table S4. Correlation between the 6-mRNA signature-based classification and clinicopathologic characteristics in 563 PDAC patients involved in the study.

Features	Low-risk Group	High-risk group	*P* value
**TCGA test series(N=181)**	**N=125**	**N=56**	
Age(mean±SD)	65.32±10.65	62.95±11.68	0.181[a]
Gender			
Female	55(44.00%)	26(46.43%)	0.762[b]
Male	70(56.00%)	30(53.57%)	
Tumor location of Pancreas			
Head	96(83.48%)	43(78.18%)	0.697[b]
Body	9(7.83%)	6(10.91%)	
Tail	10(8.70%)	6(10.91%)	
Histologic grade			
G1	26(20.97%)	4(7.27%)	0.043[b]
G2	67(54.03%)	30(54.55%)	
G3	29(23.39%)	21(38.18%)	
G4	2(1.61%)	—	
TNM stage			
I	16(13.01%)	5(8.93%)	0.468[b]
II	102(82.93%)	47(83.93%)	
III	3(2.44%)	1(1.79%)	
IV	2(1.63%)	3(5.36%)	
2-year OS status			
Alive	81	15	<0.001[b]
Dead	44	41	
**GEO batch validation series(N=251)**	**N=174**	**N=77**	
Age(mean±SD)	64.07±12.08	63.90±9.80	0.967[a]
Gender			
Female	31(49.21%)	7(38.89%)	0.439[b]
Male	32(50.79%)	11(61.11%)	
Histologic grade			
G1	2(5%)	—	0.004[b]
G2	26(65%)	6(25%)	
G3	12(30%)	17(70.83%)	
G4	—	1(4.17%)	
TNM stage			
I	13(15.66%)	4(8.89%)	0.327[b]
II	62(74.70%)	32(71.11%)	
III	5(6.02%)	6(13.33%)	
IV	3(3.61%)	3(6.67%)	
2-year OS status			
Alive	85	18	<0.001[b]
Dead	89	59	

ICGC validation series(N=96)	N=70	N=26	
Age(mean±SD)	66.37±10.92	66.96±12.09	0.820[a]
Gender			
Female	36(51.43%)	11(42.31%)	0.427[b]
Male	34(48.57%)	15(57.69%)	
Tumor location of Pancreas			
Head	57 (81.43%)	19 (73.08%)	0.458[b]
Body	4 (5.71%)	1 (3.85%)	
Tail	9 (12.86%)	6 (23.08%)	
Histologic grade			
G1	1(1.45%)	—	0.300[b]
G2	45(65.22%)	11(45.83%)	
G3	22(31.88%)	12(50%)	
G4	1(1.45%)	1(4.17%)	
TNM stage			
I	7(10.00%)	2(7.69%)	0.371[b]
II	58(82.86%)	22(84.62%)	
III	—	1(3.85%)	
IV	5(7.14%)	1(3.85%)	
2-year OS status			
Alive	36	6	0.013[b]
Dead	34	20	
**Fudan validation series(N=35)**	**N=17**	**N=18**	
Age at diagnosis, years			
≤60	11	6	0.063
>60	6	12	
Gender			
Female	9	7	0.40
Male	8	11	
TNM stage			
I	4	4	0.62
II	8	7	
III	3	6	
2-year OS status			
Alive	15	11	0.067
Dead	2	7	

—: Without available data; [a]Student's test; [b]Chi-square test.
